# Highly Dissipative Materials for Damage Protection against Earthquake-Induced Structural Pounding

**DOI:** 10.3390/ma14123231

**Published:** 2021-06-11

**Authors:** Anna M. Stręk, Natalia Lasowicz, Arkadiusz Kwiecień, Bogusław Zając, Robert Jankowski

**Affiliations:** 1 Faculty of Civil Engineering, Cracow University of Technology, 31-155 Cracow, Poland; akwiecie@pk.edu.pl (A.K.); bozajac@pk.edu.pl (B.Z.); 2 Faculty of Civil and Environmental Engineering, Gdansk University of Technology, 80-283 Gdansk, Poland; natalia.lasowicz@pg.edu.pl (N.L.); jankowr@pg.edu.pl (R.J.)

**Keywords:** polyurethane, metal foam, polymer–metal composite, structural pounding, earthquakes

## Abstract

It is a common situation that seismic excitations may lead to collisions between adjacent civil engineering structures. This phenomenon, called earthquake-induced structural pounding, may result in serious damage or even the total collapse of the colliding structures. Filling the gap between two buildings erected close to one another by using visco-elastic materials can be considered to be one of the most effective methods to avoid seismic pounding. In this paper, a new polymer–metal composite material made of polyurethane and closed-cell aluminum foam is proposed as a pounding energy absorber for protection against earthquake hazards. The composite was created in two versions, with and without an adhesive interface. A series of experiments which reflect the conditions of seismic collision were performed: quasi-static compression, dynamic uniaxial compression and low-cycle dynamic compression with 10 loops of unloading at 10% strain. The composite material’s behavior was observed and compared with respect to uniform material specimens: polymer and metal foam. The experimental results showed that the maximum energy absorption efficiency in the case of the new material with the bonding layer was improved by 34% and 49% in quasi-static and dynamic conditions, respectively, in comparison to a sole polymer bumper. Furthermore, the newly proposed composites dissipated from 35% to 44% of the energy absorbed in the cyclic procedure, whereas the polymer specimen dissipated 25%. The capacity of the maintenance of the dissipative properties throughout the complete low-cycle loading was also satisfactory: it achieved an additional 100% to 300% of the energy dissipated in the first loading–unloading loop.

## 1. Introduction

### 1.1. The Issue of the Pounding of Structures due to Seismic Load, and the Existing Solutions

Earthquakes are considered to be the most unpredictable and dangerous dynamic loads that can act on civil engineering structures [1,2,3]. Moreover, it is a very common situation that collisions between adjacent structures occur during seismic excitations due to their differences in structural dynamic parameters and the small gaps between them [4,5]. In most cases, the differences in mass or stiffness of neighboring buildings result in out-of-phase vibrations leading to collisions between them [6]. This phenomenon, called earthquake-induced structural pounding, appears when the relative structural displacement exceeds the in-between separation distance [7,8,9]. It was observed in the past that such situations may result in a large amount of damage or even collapses of neighboring structures [10,11,12]. During the earthquake in Mexico City in 1985, for example, structural pounding was one of the main causes of major damage [10]. Furthermore, after the Loma Prieta earthquake in 1989, the destructive influence of collisions was observed in more than 500 buildings that were located over 90 km from the epicenter [11]. Other examples of earthquakes causing structural damage due to pounding were summarized by Anagnostopoulos [13].

The insufficient separation between two adjacent structures increases the probability of structural pounding [14,15]. In the case of newly constructed buildings, an appropriate gap size between them has to be provided so as to avoid structural interactions during earthquakes. The research on the determination of the optimum separation distance to prevent pounding between buildings during earthquakes has recently been advanced (see [16,17,18,19] for example). However, such methodology cannot be considered in the case of existing structures. Moreover, nowadays, engineers tend to construct as many buildings as possible in the smallest possible area, hence ensuring that an appropriate in-between distance is very difficult and expensive.

It was confirmed in [20] that the application of polymer elements placed between structures may prevent damaging collisions during earthquakes, which means that the approach can be considered as an effective pounding mitigation technique. It should also be underlined that, in the case of structures erected in seismic areas, the aspect of structural damping is very important, and innovative solutions using dissipative viscoelastic elastomers like PolyUrethane Flexible Joints (PUFJ) and Fibre-Reinforced PolyUrethane (FRPU) meet these requirements (see [21,22,23,24]).

Another approach that can be considered to reduce the negative effects of pounding between insufficiently separated structures is filling the gap using the already mentioned viscoelastic materials ([20]) in the form of composite materials, either externally bonded [25,26] or injected in the cracks [27]. These types of materials have been proposed as an innovative strengthening or repair solution in masonry or infill structures [21,28,29]. Another type of composite for the prevention of earthquake damage consisting of elastomer and steel was reported in [30]. The idea of composites of polymer and metal in the function of dampers is known for combining the individual properties of each of the substrates and is also exploited in other applications, for example [31,32].

### 1.2. The Proposition of New Materials as Earthquake Damage Protection

In this paper, a new type of damping polymer–metal composite is proposed for potential anti-seismic damage purposes in buildings: a combination of polyurethane and aluminum closed-cell foam. The presented primary study is based on the carried-out fundamental research aiming at the verification of an innovative idea of seismic protection against pounding effects.

Polyurethanes are well described in already mentioned works, e.g., [20,22,23,24]. With respect to the proposed application in civil engineering, it is worth adding that materials from the family of polyurethanes are resistant to repeated hydration and repeated freeze–thaw cycles, as is shown in, e.g., [33], which describes the results of practical research conducted for airport aprons, or in paper [34], which presents the results of testing a polyurethane family member in the presence of water.

Cellular metals are materials which, due to their combination of lightweight, structural and morphological properties, find applications in various civil engineering fields [35,36,37], among which we can include architectural design, and at the same time control [38] or structural elements [39]. In other usage domains, like transportation [40] or military industry [35], cellular metals are chosen as crush absorbers and impact protection [41]. In terms of the durability of aluminum foam in hydration/dehydration cycles, the research is very scarce. On the other hand, aluminum foam spontaneously undergoes superficial oxidation and thus a natural coating appears, which is stable in the pH range 4.5÷8.5 [42]. This provides considerable protection against corrosion, because “the pH of natural waters typically lies in the range of 4.5 to 8.5” [43]. In case more protection against aggressive environmental influence would be needed, there are techniques to cover aluminum foam in an additional coating [44]. Furthermore, nondestructive methods—which are important in the case of existing structural applications—for monitoring aluminum foam’s health in terms of corrosion are being developed [45].

In this work, it is examined whether the excellent energy absorbing properties of metal cellulars could be transferred to civil engineering applications in earthquake damage protection. Typically, in energy-absorbing and impact investigations, uniaxial compression tests are performed, and plateau stress and energy absorption efficiency are determined based on the gathered stress–strain results [46]. First, energy absorption should be defined in the following Equation (1):

(1) $$W=\frac{1}{100}\int_{0}^{\varepsilon }\sigma d\varepsilon$$

where *σ* is stress (N/mm^2^), *ε* is strain (%), and W is the volumetric energy absorption (MJ/m^3^).

Now, the energy absorption efficiency can be described by Equation (2):

(2) $$W_{eff}=\frac{W}{\sigma \cdot \varepsilon }\cdot 10^{4}$$

where *σ* is stress (N/mm^2^), *ε* is strain (%), and *W* is the volumetric energy absorption (MJ/m^3^).

Energy absorption efficiency is based on the accumulative energy absorption, but it gives new information on how far from the ideal damping body ($W_{eff}^{ideal}$ = 100%) the material at the certain strain level is.

Another remark about absorbed energy should be made. In a general case of compressive experiments, one could differentiate three components of absorbed energy as is shown in the Equation (3):

(3) $$W=W_{\mathrm{el}.\mathrm{lin}}+W_{\mathrm{el}.\mathrm{nonlin}}+W_{\mathrm{diss}},$$

where $W_{\mathrm{el}.\mathrm{lin}}$ is the potential energy of elastic linear strain, $W_{\mathrm{el}.\mathrm{nonlin}}$ is the potential energy of elastic nonlinear strain, and $W_{\mathrm{diss}}$ is the dissipated energy (plastic deformation, friction, heat).

Equation (3) in the case of the presented study is simplified by the substrate of the energy of linear elastic strain. This is justified by three main factors: Firstly, polyurethanes which are going to be used in the research are visco-elastic materials which undergo almost completely nonlinear deformation. As for the other composite component, metal foam, some authors assume that the first material response in compression could be regarded as a linear elasticity regime connected with wall bending (e.g., [47]), while others observed in their research that the first deformation was only partially reversible (elastic) [48]. Lastly, for many materials, compression causes considerable plastic stain (in opposition to tensile tests) [49]. Having this in mind, it can be assumed for the present research that the potential energy of elastic linear strain is negligible, $W_{\mathrm{el}.\mathrm{lin}}\approx 0$, and that the potential energy of elastic nonlinear strain can be regarded as a total elastic component, $W_{\mathrm{el}}=W_{\mathrm{el}.\mathrm{lin}}+W_{\mathrm{el}.\mathrm{nonlin}}\approx W_{\mathrm{el}.\mathrm{nonlin}}$. In this way, the following Equation (4) for absorbed energy is obtained:

(4) $$W=W_{\mathrm{el}}+W_{\mathrm{diss}}$$

where $W_{\mathrm{el}}$ is the potential energy of elastic strain equal to the potential energy of elastic nonlinear strain and $W_{\mathrm{diss}}$ is the dissipated energy (plastic deformation, friction, heat).

Closed-cell aluminum solid foam has been widely researched in terms of static and dynamic compression, e.g., [50,51]. Previous works have shown that cellular aluminum with closed cells is sensitive to the strain rate in compression [52,53]. Moreover, article [54] combines a discussion on strain rate sensitivity analysis and energy absorption in closed-cell aluminum foams. Based on these studies, the comparison of two strain rates (simulating quasi-static and dynamic loading) is carried out in the experimental procedure of polymeric and metal materials described in the present article.

At this point, it is worth mentioning that other material properties or experimental conditions might also influence the investigated behavior. Study [55], for example, explored the dependence of the strain rate, foam density and plateau stress. Paper [56] reflected on the effects of cell-size on energy absorption in closed-cell aluminum. Work [57] touched the problem of gluing aluminum foams in composites. In the case of the present experiments, the issue of density and cell size influence can be neglected due to the usage of uniform stochastic metal foam. As for the gluing, one of the researched composites consisted of two types of layers which were connected by an adhesive layer.

### 1.3. Contributions of the Present Work

This paper presents the results of an experimental investigation concerning the potential usage of a new polymer–metal composite for filling the in-between gap in the case of buildings prone to earthquake-induced structural pounding. For this purpose, four sample types were used: uniform polymer (polyurethane Sika PST), metal foam (closed-cell aluminum) and two different composites of them combined. The first type consisted of three layers: Sika PST polymer + metal foam + Sika PST polymer. In the other one, an additional adhesive of PS polymer (polyurethane Sika PS) was used so as to connect the structural layers, ensuring that they acted in unity. The details of the used materials and samples are described in Section 2.1 and Section 2.2.

The experimental investigation was divided into three parts. In the first stage of the study, a quasi-static compression test was conducted. In a further step, a uniaxial dynamic test was performed. The third experimental procedure consisted of a dynamic low-cycle test with 10 loops of unloading at 10% strain. The results of the experimental study are presented and discussed thoroughly in Section 3. Three main criteria were chosen in order to assess the protective capacity of the new materials: their energy absorption efficiency, dissipated energy share quotient and quotient of accumulated normalized dissipated energy (defined as above, and as in Section 3.3.2). Furthermore, additional indicators such as compressive strength, energy absorption and the analysis of deformation and failure paths from stress–strain plots were used to judge the potential of the new composites in earthquake damage prevention applications.

The conducted research proved that it is possible to produce the designed composites with the bonding layer. Moreover, the assumed experimental procedure turned out to provide the necessary data for proper assessment, and the adopted measures and indicators were appropriate for reliable comparison between the studied materials. The main conclusion was successfully drawn that, in terms of the assumed criteria, the newly proposed material solution against seismic pounding has improved properties with respect to sole polymer use.

## 2. Experiments

The experimental study consisted in compression with two strain rates: 10 × 10^−3^ s^−1^ (quasi-static) and 0.2 s^−1^ (dynamic). The dynamic tests were performed as uniaxial compression (to the testing machine limit) and cyclic compression with 10 cycles of unloading at 10% strain. There were four types of samples: two composites of different structures, a uniform polymer specimen and a uniform metallic sample. The two last types played the role of the reference, with the purpose of showing how the composite structure influences potential applications in comparison to the single material use. The samples were cubic and had dimensions of 5×5×5 cm^3^.

### 2.1. Materials

The structural metallic parts of composites and the metallic reference samples were made of closed-cell aluminum foam. As for the foam’s morphological characteristics, it was closed-cellular, stochastic and isotropic (in representative volume). The material’s apparent density was equal to (0.25±0.03) g/cm^3^. The mechanical properties of the metallic foam were: compressive strength $\sigma_{c}=\left(1.45\pm 0.19\right)$ MPa, plateau stress $\sigma_{\mathrm{pl}}=\left(1.51\pm 0.19\right)$ MPa and plateau end $\varepsilon_{\mathrm{pl}.f}=\left(45.18\pm 3.15\right)\%$, all self-determined [58] according to [59,60,61]. According to a linear formula originally applied to ceramics in [62] and then applied to closed-cell aluminum in [63], the Poisson’s ratio for the used aluminum foam could be estimated as $\nu_{\mathrm{foam}}\approx 0.18$.

Polymer Sika PST was used as reference samples and structural parts in composites. According to the data produced by a uniaxial tensile test, the elastic modulus, strength and ultimate elongation of the polymer were 10 MPa, 2.9 MPa and 60%, respectively. The polymer which was used for the bonding was Sika PS. The elastic modulus, strength and ultimate elongation of Sika PS were 16 MPa, 2.5 MPa and 40%, respectively, obtained from the producer in a uniaxial tensile test. The Poisson ratio for the used polymers could be assumed to be $\nu_{\mathrm{polymer}}\approx 0.50$.

### 2.2. Samples

There were four types of samples: a uniform polymer, uniform metallic, composite type 1 and composite type 2. The metallic samples and metallic parts of the samples were machined using a stationary electric saw. The reference polymer specimens were prepared in our own laboratories from SIKA substrates. The composite samples were self-designed and their manufacturing was performed in our own laboratories. All of the specimens were cubic, with a side length of 5 cm; the specific dimensions of the samples are shown in Table 1.

#### 2.2.1. Polymeric Reference Samples

**Figure 1:**
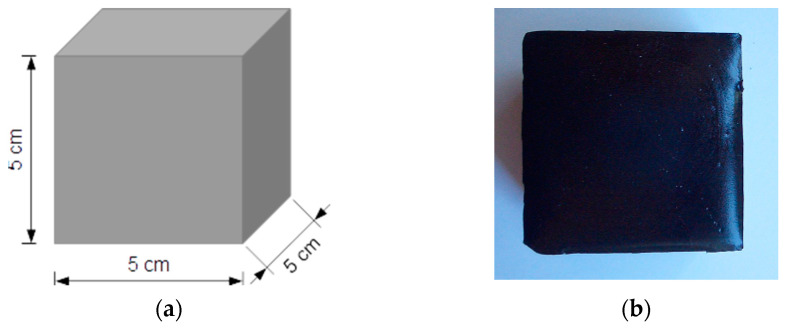
Polymeric sample: scheme (**a**) and photograph (**b**).

Three polymeric reference samples were prepared: one for quasi-static experiment (X_P_01), one for the dynamic test (X_P_02) and one for cyclic loading (X_P_03). Sika PST polymer was used to manufacture them. Figure 1a presents a schematic drawing of PST specimens, and Figure 1b shows a photograph of one of the samples (X_P_01).

#### 2.2.2. Metallic Reference Samples

**Figure 2:**
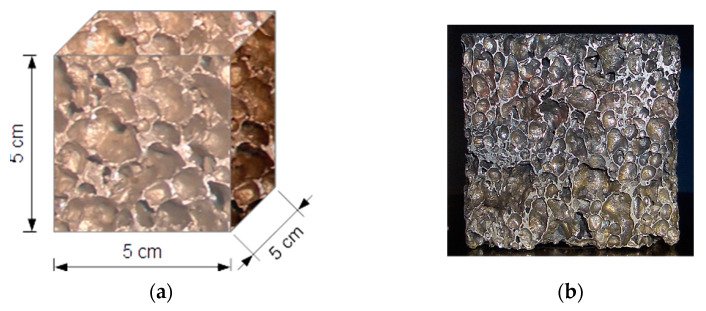
Aluminum foam sample: scheme (**a**) and photograph (**b**).

Three metallic reference samples (X_Z_01, X_Z_02, X_Z_03) were prepared by cutting them from an aluminum closed-cell material slab. The samples were used, respectively, in three assumed procedures of compression: quasi-static, simple dynamic and dynamic with loops. Figure 2 depicts a schematic drawing as well as a photograph of a sample (X_Z_02).

#### 2.2.3. Composite Type 1 Samples

**Figure 3:**
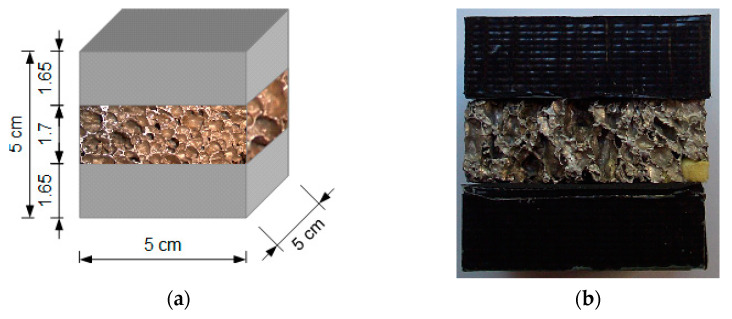
Composite type 1 sample: scheme (**a**) and photograph (**b**).

The first composite type was designed as a set of three layers: Sika PST polymer + metallic foam + Sika PST polymer. There was no adhesive nor any lubricant layer between them in order to allow for potential mutual displacement in compression tests due to only naturally occurring constraints (resulting from friction). The three layers had similar heights. Figure 3 depicts a scheme and a photograph of one of the samples (X_ZP_01). Three samples were used in the tests: X_ZP_01, X_ZP_02 and X_ZP_03 (for three types of compression tests).

#### 2.2.4. Composite Type 2 Samples

**Figure 4:**
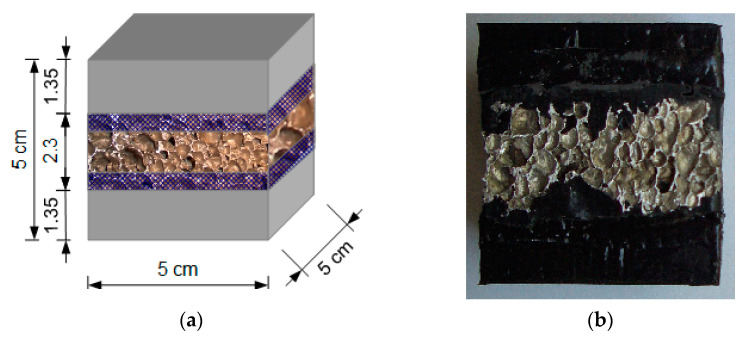
Composite type 2 sample: scheme (**a**) and photograph (**b**).

The other composite was designed as a set of three structural layers connected by an adhesive. The two outer structural parts were made of Sika PST polymer and the middle layer was made of the aluminum foam. Sika PS polymer was used as the adhesive. The adhesive layer connected the parts in a firm yet flexible way, ensuring that they act in unity. The middle metallic layer was slightly wider than the outer shells. Figure 4 depicts a scheme and a photograph of one of the samples (X_ZPS_01). Five samples were prepared: X_ZPS_01, used in the quasi-static compression; X_ZPS_02 and X_ZPS_04, used in the dynamic simple compression; and X_ZPS_03 and X_ZPS_05, which were subject to loop loading.

The characteristics of the individual samples are presented in Table 1. The following symbols were assumed to characterize the samples: *a* and *b* stand for the dimensions of the cross-section, while *h* is the height. Moreover, P stands for polymeric specimens, Z is for uniform aluminum foam specimens, ZP denotes composite samples without the adhesive layer, and ZPS represents samples with the adhesive layer present.

### 2.3. Experimental Procedure

For all of the samples, except for sample X_Z_01, the experiments were performed using Zwick 1455 testing machine (ZwickRoell GmbH & Co. KG, Ulm, Germany) with 20 kN capacity, and using the computer programme TestExpert III (ZwickRoell GmbH & Co. KG, Ulm, Germany, version 1.2). Sample X_Z_01 was tested in an MTS 810 testing machine (MTS Systems Corporation, Eden Prairie, MN, USA) with an additional force sensor Interface (capacity: 25 kN), and with the use of the computer programme TestWorks4 (MTS Systems Corporation, Eden Prairie, MN, USA, version V4.08D). Both of the testing machines were of class 1. Photographical documentation in all cases was gathered with Casio Exilim EX-Z55 (Casio Computer CO., LTD, Tokio, Japan).

The experiments consisted in uniaxial compression tests with two different strain rates. In the first case, the tests were quasi-static (strain rate 10 × 10^−3^ s^−1^) and in the second case they were dynamic (strain rate 0.2 s^−1^). The final load was assumed to be 24 kN in the quasi-static experiments, while in the dynamic experiments it was 20 kN. In both cases, the initial stress (preload stress) was set as 0.01 MPa. The dynamic testing was performed in two modes: uniaxial compression and compression with cyclic unloading (10 loops) at 10% strain. All of the tests were conducted at room temperature.

**Figure 5:**
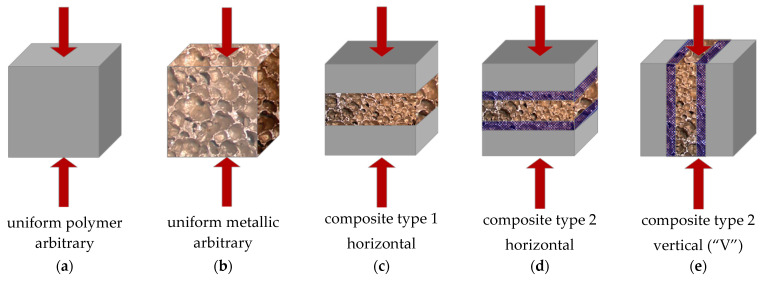
Orientation of the sample types with respect to the load direction.

The reference polymer and aluminum specimens were isotropic; hence, their orientation with respect to load direction was arbitrary. The composite samples were layered, so their orthotropy influenced their compressive behavior. Taking this fact into account, certain sample positions were assumed with regard to the loading direction; they are schematically shown in Figure 5.

## 3. Results and Discussion

**Figure 6:**
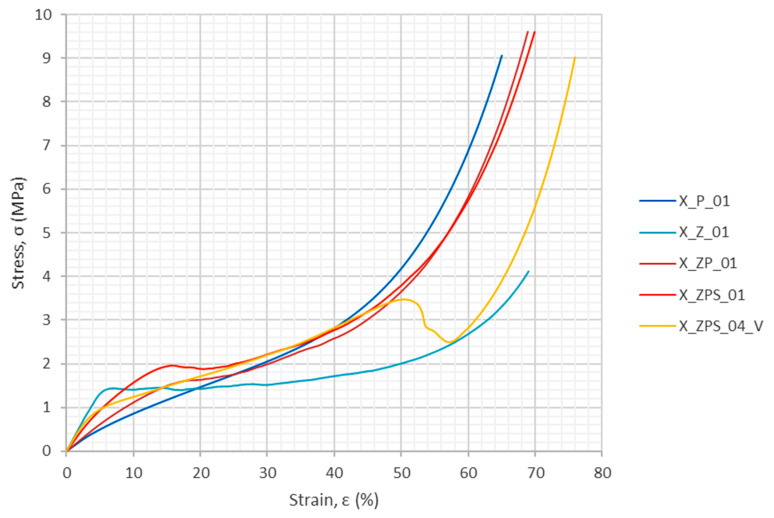
Stress–strain curves from quasi-static experiments.

**Figure 7:**
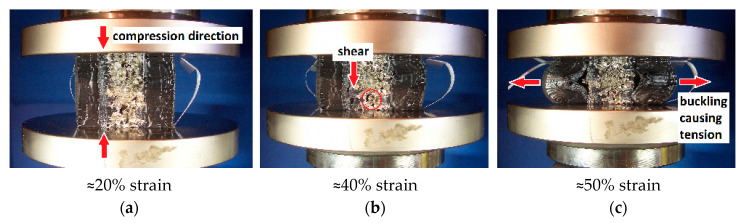
Deformation of the bonding layer of specimen X_ZPS_04_V at different strain levels: (**a**) at ≈20% strain, the arrows show the direction of the overall compressive forces in the bonding layer; (**b**) at ≈40% strain, the arrows show a region of collapsed cells and detachment, possibly due to shearing in the nearby foam core; (**c**) at ≈50% strain, the arrows show the delamination and distinct bulking of the outer layer, causing tensile forces.

**Figure 8:**
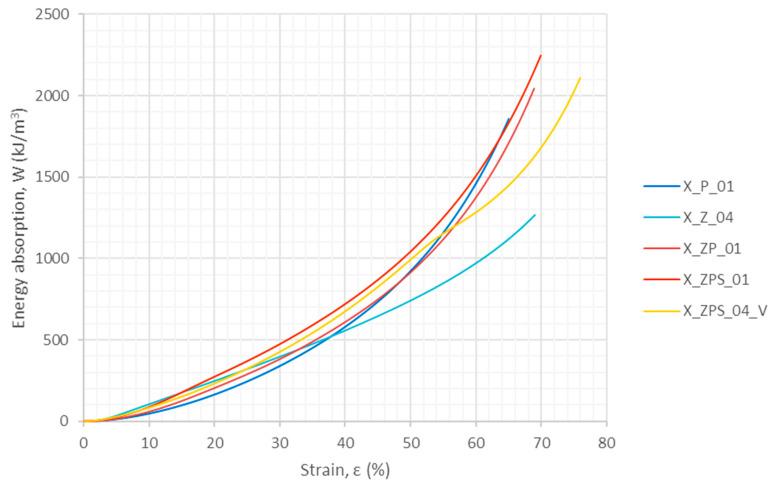
Energy absorption in the function of the strain from the quasi-static experiments.

**Figure 9:**
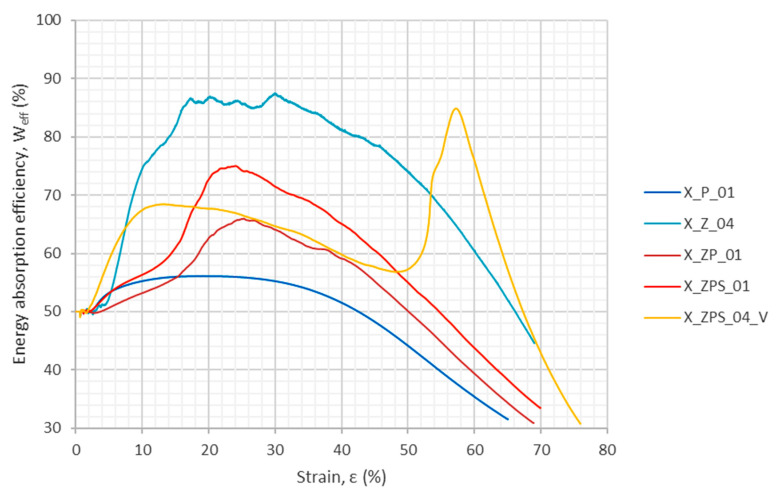
Energy absorption efficiency as a function of the strain from the quasi-static experiments.

**Figure 10:**
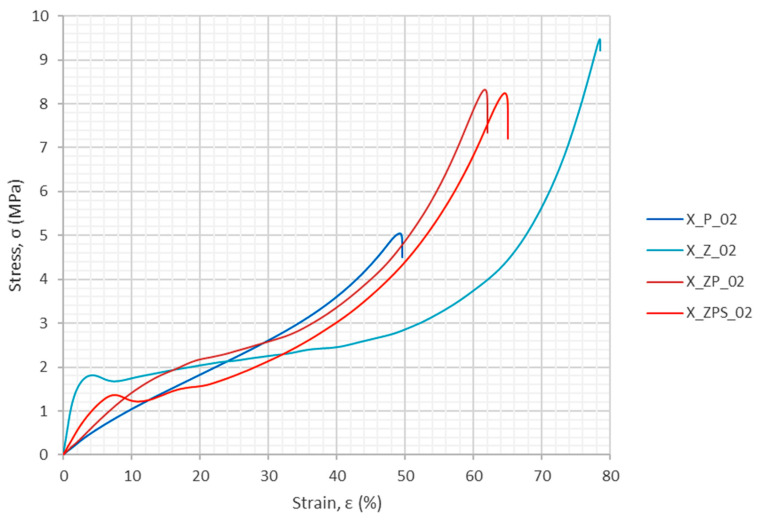
Stress–strain curves from the dynamic experiments.

**Figure 11:**
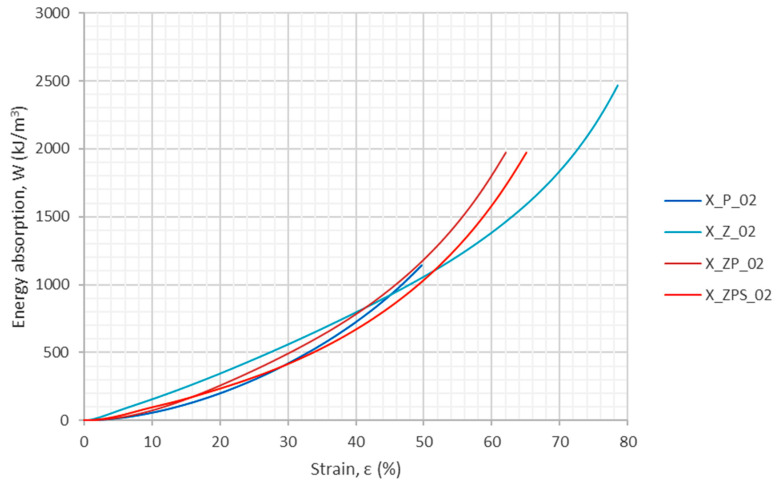
Energy absorption as a function of the strain from the uniaxial dynamic experiments.

**Figure 12:**
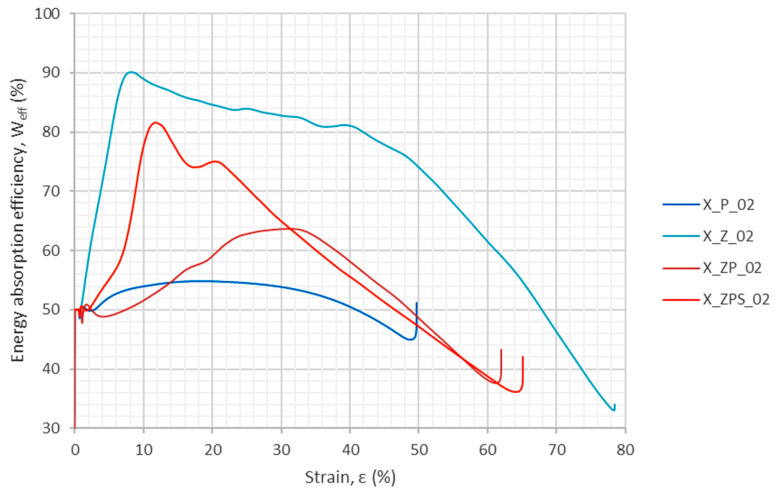
Energy absorption efficiency as a function of the strain from the uniaxial dynamic experiments.

**Figure 13:**
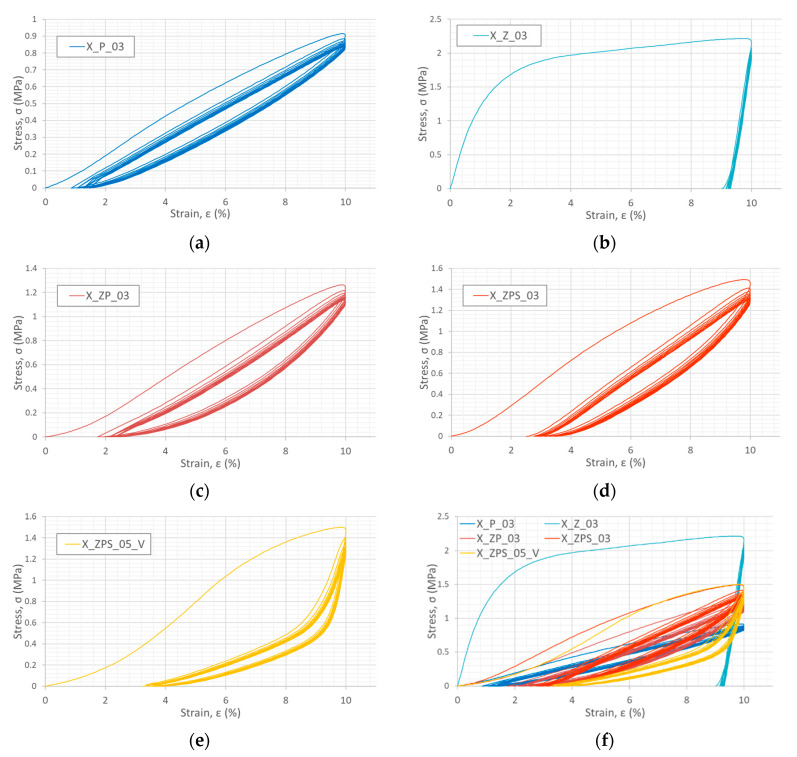
Stress–strain graphs from dynamic compression with cyclic unloading for individuals specimens: X_P_03 (**a**), X_Z_03 (**b**), X_ZP_03 (**c**), X_ZPS_03 (**d**), X_ZPS_05_V (**e**) and for the comparison of stress magnitude also for all samples plotted together (**f**).

**Figure 14:**
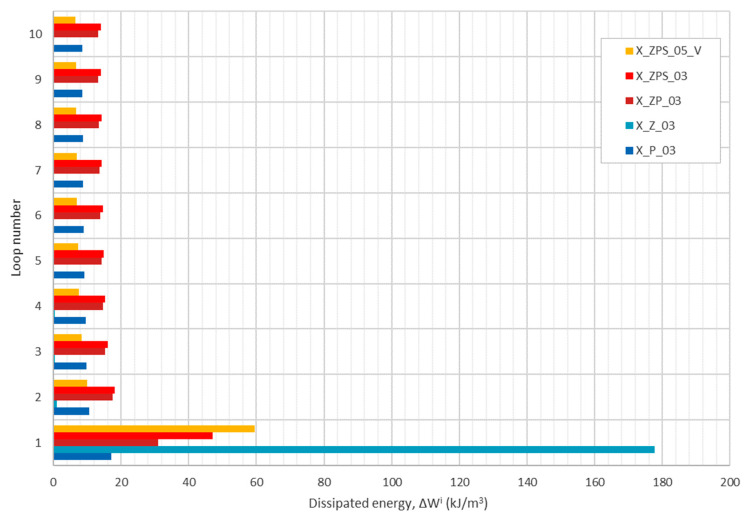
Dissipated energy according to the unloading cycle for all of the tested specimens.

The data gathered in the experiments are presented below in the form of diagrams: Figures 6–9 are for quasi-static tests, Figures 10–12 are for dynamic uniaxial compression, and Figures 13 and 14 are for the trials with loops. Furthermore, the characteristic measures for each specimen type were determined and are summarized in Table 1, and the numerical results from the experiments are set in Tables 2–6.

### 3.1. Quasi-Static Compression

The following five samples were chosen for these tests: X_P_01, X_Z_01, X_ZP_01, X_ZPS_01 (horizontal) and X_ZPS_04 (vertical). It should be noted that at around 40% strain, delamination occurred in sample X_ZPS_04 and it lost its integrity.

#### 3.1.1. Stress–Strain Results from Quasi-Static Compression

Figure 6 depicts the experimental stress–strain diagram for all five types of specimens studied in quasi-static compression. For all of the stress–strain curves, a “zero point” was assumed as the intersection of the horizontal axis with the extension of the first linear segment of the graph. The uniform polymer sample (X_P_01) has the smoothest curve; on the other hand, the uniform aluminum foam sample (X_Z_01) has the most distinct plateau region and the first local maximum at its beginning. The three remaining sandwich-type specimens inherit the curve steepness characteristic for the polymer and combine it with the distinct change of compressive response in the neighborhood of the initial local maximum, as it is for the metal foam. As was mentioned above, X_ZPS_04 underwent delamination at a strain above 40%; however, the cross-section area was retained in the calculation of the initial stress (for the integrated sample) and not as for three parts acting separately. The laminar destruction of the sample is visible in the distinct drop at a strain of 50–60% for this specimen in the stress–strain diagram (see Figure 6).

Based on the stress–strain data, one can determine the compressive strength. Depending on the shape of the initial stress–strain graph, this magnitude is defined in two ways [61]:

- $\sigma_{c}$—the stress which is the first local maximum in the stress–strain graph (the first maximum compressive strength);
- $\sigma_{c}^{*}$—in the case of no local maximum, one determines the intersection of the extensions of the first two linear sections of the stress–strain graph; the ordinate of the intersection point is the compressive strength.

Compressive strength, defined in such a way, is characteristic for materials which undergo plastic collapse. Therefore, this magnitude was determined for the uniform aluminum sample and for the composites, see Table 2. Additionally, the strain for which the compressive stress occurred was also found, because it informs one about the range of the first material response type. The results given in Table 2 show that in the uniform metal foam sample, plastic collapse started at the earliest stage, at about 7% strain (and at the stress of about 1.5 MPa). The presence of polymer layers in the analyzed composites with horizontal orientation caused considerable lengthening of the first response region, up to about 16% strain (the respective strength ≈ 2 MPa) and about 18.5% strain (the respective strength: ca. 1.6 MPa). The composite with vertical layers cannot be compared straightforwardly with the latter results, because there was no distinct first local maximum of the stress obtained in this case, as it was in other specimens.

It is worth taking more insight into the character of the first regime now. Some authors assume that the first metal foam response in compression could be regarded as a linear elasticity regime connected with wall bending (e.g., [47]). However, others observed in their research that the first deformation was only partially reversible [48], implying that plastic deformation starts before reaching stress equal to the compressive strength. On the other hand, polymers are widely known to exhibit viscoelastic (nonlinear elastic) behavior even up to large deformations [64]. From the observed results, it can be inferred that the combination of aluminum foam with polymer in the form of a layered composite allows one to obtain a larger range of the first regime and add a considerable elastic component to it.

This, however, comes at a cost, which is the absence of the plateau in the composites. The plateau in the stress–strain graph of the aluminum sample represents plastic deformation and a regime called densification, which is caused by consequent individual cells collapsing. This deformation mode is responsible for the excellent energy absorption property of metal foams. Some of this feature it traded for the adding and/or lengthening of the elastic regime by adding a polymer in the studied composites.

The delamination observed in specimen X_ZPS_04_V during the quasi-static compression is shown in a few stages in Figure 7. The process developed gradually: at 20% strain, only a little transversal bulk deformation in the middle zone of the specimen was visible; at 40% strain, the delamination started in the central regions of the bond between the foam core and the adhesive layer; finally, at 50% strain, the distinct detachment of the adhesive at the edge of the foam appeared. Such a manner of delamination is a result of the superimposed effects of a few phenomena, which are now going to be discussed in their turn.

For a start, the morphological characteristics of the bonding zone are complex. The middle layer (metal) had a rough surface of open cavities (cells cut open due to the machining of the specimen). On the other hand, the surface of the external polymer was smooth. The bonding Sika PS was made to penetrate the superficial cavities of the metal core and adhere strictly to the polymer shell; thus, the layer was interlocking, with half-bubbles at the inner verge interspersed with aluminum cell walls. Additionally, there was a considerable difference in the Poisson’s ratio of the component materials, which caused them to deform in two different modes. For the aluminum foam, the value might be assumed to be $\nu_{\mathrm{foam}}\approx 0.18$, causing a relatively small bulk effect due to cells partially collapsing inwards during squashing. For the polymers, of which the Poisson’s ratio could be estimated as $\nu_{\mathrm{polymer}}\approx 0.50$, the bulking effect is significant due to their considerable incompressibility. Furthermore, for the polymer shell, the bond was unilateral (its external verge was the external verge of the sample) and for the core, the bond was on its both sides. Lastly, one must take into account the different friction of the materials with respect to the machine presses.

Taking all of the above mentioned factors into consideration, the description of the failure in the adhesive layer requires more in-depth research to be complete. Nevertheless, based on the identification of these factors, one can infer that at least three failure-inducing mechanical phenomena occur in the bond: 1/ compression due to the overall testing compressive character, but also ‘transmitted’ due to the interlocking aluminum cell walls (see Figure 7a); 2/ the shearing of the bonding polymer resulting from individual metal cells collapsing (see Figure 7b); 3/ the tensile action of the bulking outer polymer layers due to their incompressibility, and thus their tendency to widen towards the bond-free edge (see Figure 7c).

#### 3.1.2. Energy Absorption in Quasi-Static Compression

The energy absorption from the quasi-static experiments was calculated using Equation (1). The results are shown in Figure 8. All of the curves are similar in shape; however, this remark does not apply ideally to the specimen with vertical layers, due to the delamination, and to the uniform aluminum foam, due to the distinct first maximum stress. The nonlinearity of the relationship of the absorbed energy with respect to strain is visible.

Figure 9 depicts the energy absorption efficiency as a function of the strain from the quasi-static experiments; the results shown here were calculated with the use of Equation (2). It can be noticed that, in the case of the polymer sample, no distinct extremum is visible and the curve is the lowest one. On the other hand, aluminum foam is the top line, with a series of local maxima $W_{eff}\in$ (84; 88)% for strains ε∈ (15; 30)%, which more or less corresponds to the plateau region. Again, the sandwich specimens combine the features of their contributors, and one can see the lower overall curse of curves with a single distinct maximum (the second maximum for X_ZPS_04_V is attributed to the drop in the stress–strain curve caused by delamination).

Energy absorption is an accumulative measure; hence, it is convenient to compare the ways in which the studied materials behave at certain fixed strain levels which can be chosen according to the designed application demands. In the present research, the energy absorption *W* was specifically determined for the following strain values: 10%, 20%, 30% and 40%. If all of the tests had ended at the same deformation level (e.g., a strain equal to 70%), the total energy absorption would also have been a valuable outcome of the research; however, the studied samples underwent disintegration at such different strain levels that the comparison of their total energy absorption does not add significant information. The energy absorption efficiency *W_eff_* was especially calculated for the same fixed strain levels as above: 10%, 20%, 30% and 40%. Additionally, the maximum energy absorption efficiency was determined together with the strain value for which it occurred.

The results are given in Table 3. For the purpose of easier comparison between the uniform materials and the composites, the relative values of the energy absorption and its efficiency are given in each row in the bottom line in brackets. The relative values were determined with respect to the results obtained for the uniform polymer specimen, and are without a specific unit. One can observe that, for sample X_ZPS_01, the maximum energy absorption efficiency was improved by 34%.

### 3.2. Dynamic Uniaxial Compression

The following samples were tested in uniaxial dynamic compression: X_P_02, X_Z_02, X_ZP_02 and X_ZPS_02 (horizontal).

#### 3.2.1. Stress–Strain Results from the Dynamic Uniaxial Compression

The experimental stress–strain diagrams for the dynamic uniaxial compression are shown in Figure 10. Similarly to the quasi-static tests, there was a “zero point” determined for each curve. It was assumed to be at the intersection of the abscissa axis with the extension of the first straight-line segment of the data plot. The uniform polymer sample (X_P_02) has the steepest curve. On the contrary, the uniform aluminum foam (X_Z_02) has the characteristic first maximum connected with compression strength, and a long plateau region after it. The composite sample which did not have the bonding (X_ZP_02) behaved similarly to the aluminum foam, while the other complex specimen with adhesive layers (X_ZPS_02) was more like the uniform polymer.

The identification of the compressive strength in dynamic experiments was conducted in a similar way as in previous tests. This time the upper index “*d*” was added to symbols of compression strength to differentiate the dynamic character of compression:

- $\sigma_{c}^{d}$—the stress which is the first local maximum in the stress–strain graph (the first maximum compressive strength in the dynamic experiments);
- $\sigma_{c}^{d*}$—in the case of no local maximum, the ordinate of the intersection of the extensions of the first two linear sections of the stress–strain graph is the compressive strength in the uniaxial dynamic experiments.

This measure was determined for the specimens including the aluminum foam component. Furthermore, the strain for which it occurred was found. The results are presented in Table 4.

#### 3.2.2. Energy Absorption in Uniaxial Dynamic Compression

The energy absorption in the uniaxial dynamic tests was computed in the same way as in the quasi-static research, i.e., according to Equation (1). The graphical results are plotted in Figure 11. One can observe that the function of the energy absorption with respect to strain is nonlinear.

The energy absorption efficiency was calculated as defined in Equation (2). The results are depicted in Figure 12. It can be observed that the polymer specimen (X_P_02) has the flattest curve, without a specific maximum. The curve for the composite without adhesive (X_ZP_02) is located slightly above it, with a visible maximum at around 30% strain. The third plot belongs to the composite with the adhesive layer (X_ZPS_02); it shows two distinct extrema at around 12% and 20% strain. The top graph is for the aluminum foam (X_Z_02); it has a distinct first maximum and then, for a long series of strains, only a slight slope of a high efficiency level (>80%).

Specific strain values—10%, 20%, 30% and 40%—were chosen as the levels for which numerical values of energy absorption and energy absorption efficiency were determined, as shown in Table 5. Furthermore, the maximum energy absorption efficiency was found for each specimen, along with the strain for which it occurred. The total energy absorption was not determined because the experiments did not finish for all of the samples at the same strain threshold. In order to allow a more convenient comparison between the results, the relative ratio is enclosed in each table entry below the main value in brackets. The uniform polymer specimen was chosen as the reference and denoted “ratio 1.00”; the ratios are unitless. The improvement is clearly visible, as sample X_ZPS_02 gained 49% in terms of its maximum energy absorption efficiency. The comparison of the relative ratios indicates that the combination of the uniform polymer with the metallic foam layer produces a composite with advantageous dissipation properties at various high strain levels.

### 3.3. Dynamic Compression with Unloading Loops

Five samples were ordinated for the experiments with unloading loops: uniform polymer (X_P_03), uniform metal foam (X_Z_03), composite type 1 (X_ZP_03) and composite type 2, in two layer orientations: horizontal (X_ZPS_03) and vertical (X_ZPS_05). The cyclic dynamic experimental procedure was conducted in the following way: the starting point of the unloading was set as 10% strain, and the turning point for the next loading was assumed to be zero stress. The loop of loading and unloading was repeated ten times.

#### 3.3.1. Stress–Strain Results Obtained from Cyclic Dynamic Compression

The stress–strain plots for each examined sample are depicted in Figure 13a–e. Additionally, there is a multiple plot for all of the samples presented in Figure 13f for better comparison between them. The differences between the behaviors of the various types of samples are distinct.

Firstly, the maximum stress after which the initial unloading started was the greatest for the metal foam, $\sigma_{\max}=2.21$ MPa, and the lowest for the polymer, $\sigma_{\max}=0.92$ MPa. For the composite specimens, the maximum stress assumed values between 1.2 MPa and 1.5 MPa; for specific data refer to Table 6. Furthermore, the presence of the adhesive layer, both in the horizontal and vertical layer orientation, strengthened the material.

With regard to the steepness of the loops, the aluminum foam specimen has the most abruptly ascending graph, which reflects the fact that its deformation is almost completely permanent. The sample, after full unloading, reached more than 9% strain. On the other hand, the uniform polymer sample was typical of elastomers’ large capacity for reversible deformation (the strain at the bottom of the loop is even less than 1%). The composite specimens also exhibited good potential for deformation recovery: the turning loop point was between 2% and 4% strain. Interestingly, the loops of the composite with vertical layers are steep in the upper part and only slightly inclined in the bottom section. This implies that, for such an orientation of the composite material’s orthotropy axes with respect to the loading direction, the component materials take dominant roles in the sequence forced by the presence of the adhesive zone. In the first loading, the metal core undergoes predominantly irreversible deformation and then, during the unloading—only to a limited extent assured by the interface bonding—regains a very small part of its deformed height due to the forces of elastic recovery of the outer polymer shell. On the other hand, the outer layers undergo considerable return expansion in length, but are to some small degree stopped by the fact that they are glued to already-shortened aluminum foam. In this way, after the first cycle, the sample is still in unity (the bonding is not destroyed), but the core has a different size to the skin layers, and the internal load between the layers is added at interphases: compressive for the polymer, tensile for the aluminum foam and shear at the adhesive zone. In the next loops, the protruding Sika PST parts are the first to contact the presses of the testing machine, hence the initial segments of the loading loops are slightly inclined, characteristically for the elastomer. Then, only after the presses reach the core is the load subjected to the metal foam (the steeper loop part).

#### 3.3.2. Energy Absorption in Dynamic Cyclic Compression

For all of the specimens, except for the uniform metal one, the unloading and loading paths of the loops do not converge, i.e., hysteresis occurs (see Figure 13). The explanation for these results is that, in polymers, the energy from the work of compressive force on the shortening of a specimen is absorbed by the material as potential elastic strain energy during loading. In unloading, this energy is released in the regaining of the sample’s height. However, part of the energy is dissipated due to the combination of material viscoelasticity [23] and Mullin’s effect [65] or heat [66]. The loss is reflected in the hysteresis between the loading and unloading curves in the loop plot. In the case of the aluminum, almost all of the energy from the work of the load is consumed in irreversible deformation, and only a little is stored as potential elastic strain energy.

The analysis of the energy absorption for the tested specimens was performed individually for the loops from 1 to 10 and cumulatively for the whole cyclic compression process. The calculation of the absorbed energy was conducted according to Equation (1), separately for the loading and unloading curves. The difference between the energy absorbed in the loading and during the decreasing of the load is regarded as dissipated energy. The results are plotted in Figure 14 as a horizontal column diagram of dissipated energy with respect to the consecutive loop number and specimen type. The most energy was dissipated during the first cycle; however, there is a clear difference between the analyzed materials: the uniform samples obtained extreme values (aluminum was the greatest and the polymer was the lowest) and the composite materials were in the middle zone. In the next loops, starting from the second, the dissipated energy remained at a constant level. In the aluminum, there was almost no energy dissipated for all of the loops >2. In X_P_03 and X_ZP_03, the dissipation remained at the level of about 60% of the initial value. In the case of the specimens with the adhesive, the dissipation did not fluctuate between consequent cycles and remained at about 50% and 8% of the initial value for the horizontal and vertical orientations, respectively.

Apart from analysis of loops separately, we also performed calculations for the accumulative energy. According to Equation (4), the absorbed energy can be regarded as a sum of the potential elastic strain energy (recoverable) and dissipated energy (lost). In order to show the magnitude of the shares of the two components, the quotients were defined in the following Equations (5) and (6):

- the potential elastic strain energy share quotient $q_{W.\mathrm{el}}^{\mathrm{tot}}$
  (5) $$q_{W.\mathrm{el}}^{\mathrm{tot}}=\frac{W_{\mathrm{el}}^{\mathrm{tot}}}{W^{\mathrm{tot}}}\cdot 100,\;(\%);$$
- the dissipated energy share quotient $q_{W.\mathrm{diss}}^{\mathrm{tot}}$
  (6) $$q_{W.\mathrm{diss}}^{\mathrm{tot}}=\frac{W_{\mathrm{diss}}^{\mathrm{tot}}}{W^{\mathrm{tot}}}\cdot 100,\;(\%)$$
  where $W^{\mathrm{tot}}$ is the sum of the energy absorbed in all 10 loops; $W_{\mathrm{el}}^{\mathrm{tot}}$ is the sum of the potential elastic strain energy absorbed in all 10 loops and $W_{\mathrm{diss}}^{\mathrm{tot}}$ is the sum of the energy dissipated in all 10 loops.

Additionally, with the purpose of assessing how much energy can be dissipated in 10 loops with respect to the first loop, yet another measure was introduced in the Equation (7):

- the quotient of the accumulated normalized dissipated energy $q_{W.\mathrm{diss}}^{\mathrm{norm}.\mathrm{tot}}$
  (7) $$q_{W.\mathrm{diss}}^{\mathrm{norm}.\mathrm{tot}}=\sum_{1}^{i}\left(\frac{W_{\mathrm{diss}}^{i}}{W_{\mathrm{diss}}^{1}}\cdot 100\right),\;(\%)$$
  where $W_{\mathrm{diss}}^{i}$ is the energy dissipated in the *i*-th loop, $W_{\mathrm{diss}}^{1}$ is the energy dissipated in the first loop, and i is the loop number; here i=1, 2… 10.

The results of the computations are shown in Table 6. Regarding the share quotients, one can observe that the uniform polymer and metal samples have almost exactly opposite respective values. In the case of X_P_03, the dissipated energy was ca. 25% of all of the absorbed energy after ten loops, whereas for X_Z_03 it was about 73%. For the composite samples with horizontal layers, the lost energy quotient was ca. 35%, regardless of the absence or presence of the adhesive layer. In the composite with a vertical orientation, it was nearly 44%.

Taking into account the quotient of the accumulated normalized dissipated energy, it is visible that the Sika PST uniform polymer and the composite without the adhesive were capable of dissipating four to five times as much energy in loops 2 to 10 as in the first loop. On the contrary, the aluminum foam lost its capacity for dissipation after the first cycle almost totally (only 1.6% in the consecutive nine cycles altogether). The specimens with the adhesive layer caused the loss of 100% (vertical orientation) and almost 300% (horizontal layers) more in loops >2 than in the first loading and unloading procedure.

## 4. Conclusions

This paper was devoted to the experimental study concerning the effectiveness of different materials which could be used for filling the gap between two adjacent buildings so as to protect them against damage due to collisions from seismic excitations. The negative effects of pounding can be reduced by specially designed dissipaters of energy installed inside the gap. The impact energy increases when the contact time during the collision process is shortening; thus, solutions allowing for the elongation of the pounding time are sought. The visco-elastic properties of materials are used for the damping of repeated (cyclic) forces, whereas the plastic characteristics are exploited in the dissipation of non-repeatable forces. In the first case, the visco-elastic absorption ability of polymers is caused by the partially recoverable dislocation of structural particles, which allows for the repeatable absorption of energy. In the second case, the irreversible absorption ability of metal foams follows from the plastic damage of the structural skeleton. The intention of this fundamental research was to verify the possibility of combining both absorption abilities through the tested composite materials to construct an innovative dissipater. The following results were obtained and conclusions were drawn:

- As the primary effect of this research, it has been confirmed that it is possible to combine a special polyurethane material and aluminum foam with a closed-cell structure to produce composite materials of two types, with and without an adhesive layer, and that the composites can work effectively utilizing both the viscoelastic and plastic paths of deformation simultaneously, allowing for singular and repeated collision load bearing in the case of the pounding effect from seismic excitations.
- The experiments were designed as compressions with two strain rates: 10 × 10^−3^ s^−1^ (quasi-static) and 0.2 s^−1^ (dynamic); the dynamic tests were performed as uniaxial compression and compression with 10 loops of unloading at 10% strain. Such conditions were assumed due to the sensitivity of both the polymer and the aluminum foam to the speed of the loading, and in order to mimic the conditions of the seismic collisions of structures, which have a low-cycle repeated pounding character.
- A few measures were proposed to assess the effectiveness of the new composites; among them were the compressive strength, energy absorption efficiency (acc. to Equation (2)), dissipated energy share quotient (acc. to Equation (6)) and quotient of the accumulated normalized dissipated energy (acc. to Equation (7)). The energy absorption efficiency was determined for fixed strain levels, i.e., 10%, 20%, 30% and 40%, and additionally the maximum value was found. In order to appropriately estimate how the studied materials could fit the anti-pounding application, it is necessary to analyze the suggested measures altogether, not separately.
- Such an analysis was performed, and its results confirmed that composite specimens exhibited favorable values of the above indicators. Firstly, in almost all cases, the energy absorption efficiency for the composites was between the values for the uniform polymer and aluminum foam. Especially, the maximum energy absorption efficiency for the new composites in comparison to the uniform polymer specimens was enhanced by 34% and 49%, respectively, in the quasi-static and dynamic experiments. Furthermore, the dissipated energy share quotients showed that the newly proposed materials dissipated from 35% to 44% of the energy absorbed in 10 loading-unloading cycles. For comparison, the cellular metal specimen dissipated 73% and the polymer one dissipated 25% of total absorbed energy. As for the ability of retaining the dissipation capacity for more than just one loading cycle, all of the materials exhibited the trend of keeping an almost constant level of lost energy through loops 2 to 10. The layered materials with the adhesive attained a total capacity of dissipating energy between one and three times more than in the first loop; the aluminum foam only achieved 1.6%, and the polymer and the composite without the bonding achieved above four times more.

The results of the study clearly show that the proposed composite materials can be effective in preventing damaging collisions between adjacent structures during earthquakes, and that they are worthy of further, more advanced experimental research.

One of the aspects which should be examined more thoroughly is the orientation and dimensions of the layers. It is planned to examine the ways in which the widths of the core layer and the outer shell influence the studied energy-based parameters. Apart from the proportions of the layer dimensions, the presence and type of the adhesive layer should be investigated, because the outcomes for the composites with and without the bonding interface do not assure the univocal assessment of which of the three assumed solutions is the most favorable. Other aspects for further study would be how to assure the best desired protective function with respect to the relationship of the size of the new composite filling to the actual gap size between buildings and, secondly, how to provide the most favorable attachment of the material to buildings.

The present article was devoted to new composite materials intended to be applied in bumper elements which are supposed to be attached to only one structure in the gaps between adjacent structures so as to mitigate their pounding during earthquakes. Therefore, the experimental tests were focused only on the compression behavior of the materials. It is also possible to consider the proposed polymer–metal composite materials for link elements connecting two structures, which involve both compression and tension. However, such an application would require conducting further tests so as to verify the behavior of the materials under tensile forces. It is believed that some similarities in tension behavior can be expected with relation to the previously tested elements made of polyurethane alone (see [23,64]).

Lastly, along with choosing the best material, configuration attempts at modelling should be made; the research on modelling cellular aluminum has been already started by one of the authors [58]. The model is planned to use artificial intelligence to evaluate the stress–strain relationship of the foam with respect to its density. Similar work has already been performed by one of the authors for open cell aluminum: in works [67,68] there is a detailed description of the algorithm used to choose the best neural network architecture and the result of numerical calculations, i.e., the model with the mean absolute relative error assuring good engineering precision <5%. As for the polymeric material, the five-parameter Mooney–Rivlin material model can be used to simulate the behavior of polyurethane. The model is often applied for the modelling of highly nonlinear hyperelastic materials, including elastomers and rubber-like materials [69]. The Mooney–Rivlin material model is considered to be one of the most accurate models, and is therefore the most frequently used in numerical simulations [70].

## Figures and Tables

**Table 1:** Characteristics of the samples.

Sample ID	*a* (mm)	*b* (mm)	*h* (mm)
X_P_01	50.89	52.02	49.90
X_P_02	50.84	51.47	52.14
X_P_03	50.24	51.58	52.73
X_Z_01	50.29	50.11	50.17
X_Z_02	48.80	50.00	48.00
X_Z_03	49.83	49.31	49.60
X_ZP_01	50.21	49.73	51.20
X_ZP_02	49.51	50.25	52.18
X_ZP_03	49.66	49.89	53.76
X_ZPS_01	49.78	50.18	53.70
X_ZPS_02	49.94	49.50	53.10
X_ZPS_03	49.99	50.11	54.47
X_ZPS_04	49.95	50.16	52.99
X_ZPS_05	50.23	49.90	52.78

**Table 2:** Compressive strength determined in the quasi-static tests.

Measure	X_P_01	X_Z_01	X_ZP_01	X_ZPS_01	X_ZPS_04_V
$\sigma_{c}$—compressive strength (first maximum), (MPa)	---	1.44	1.62	1.96	---
$\sigma_{c}^{*}$—compressive strength (no first maximum), (MPa)	---	---	---	---	0.94
Strain for the compressive strength, (%)	---	7.09	18.52	15.77	3.66

**Table 3:** Energy absorption and energy absorption efficiency from the quasi-static compression.

Measure	X_P_01	X_Z_01	X_ZP_01	X_ZPS_01	X_ZPS_04_V
$W_{10}$—energy absorption for 10% strain, (kJ/m^3^)	47.54 (1.00)	104.96 (2.21)	59.33 (1.25)	88.30 (1.86)	83.86 (1.76)
$W_{\mathrm{eff}.10}$—energy absorption efficiency for 10% strain, (%)	55.25 (1.00)	74.53 (1.35)	53.20 (0.96)	56.34 (1.02)	67.45 (1.22)
$W_{20}$—energy absorption for 20% strain, (kJ/m^3^)	164.51 (1.00)	247.58 (1.50)	204.53 (1.24)	273.90 (1.66)	232.05 (1.41)
$W_{\mathrm{eff}.20}$—energy absorption efficiency for 20% strain, (%)	56.10 (1.00)	86.75 (1.55)	62.65 (1.12)	72.73 (1.30)	67.68 (1.21)
$W_{30}$—energy absorption for 30% strain, (kJ/m^3^)	340.00 (1.00)	696.91 (2.05)	382.35 (1.12)	474.35 (1.40)	426.75 (1.26)
$W_{\mathrm{eff}.30}$—energy absorption efficiency for 30% strain, (%)	55.23 (1.00)	87.34 (1.58)	64.06 (1.16)	71.46 (1.29)	64.68 (1.17)
$W_{40}$—energy absorption for 40% strain, (kJ/m^3^)	580.58 (1.00)	557.87 (0.96)	610.07 (1.05)	720.90 (1.24)	675.64 (1.16)
$W_{\mathrm{eff}.40}$—energy absorption efficiency for 40% strain, (%)	51.54 (1.00)	81.14 (1.57)	59.12 (1.15)	65.08 (1.26)	59.81 (1.16)
$W_{\mathrm{eff}}^{\max}$—maximum energy absorption efficiency, (%)	56.11 (1.00)	87.49 (1.56)	65.97 (1.18)	75.01 (1.34)	68.46 * (1.22) 84.89 (1.51)
Strain for the maximum energy absorption efficiency, (%)	18.36 (1.00)	29.89 (1.63)	25.23 (1.37)	24.02 (1.31)	13.26 * (0.72) 57.21 (3.12)

* For the sample X_ZPS_04_V, there were two local maxima of the energy absorption efficiency (the second in the effect of delamination). They are given in the same table entry; also, both respective strains are included in one entry.

**Table 4:** Compressive strength determined in the dynamic tests.

Measure	X_P_02	X_Z_02	X_ZP_02	X_ZPS_02
$\sigma_{c}^{d}$—compressive strength (first maximum), (MPa)	---	1.81	---	1.36
$\sigma_{c}^{d*}$—compressive strength (no first maximum), (MPa)	---	---	1.78	---
Strain for the compressive strength, (%)	---	4.22	12.45	7.51

**Table 5:** Energy absorption and energy absorption efficiency from uniaxial dynamic compression.

Measure	X_P_02	X_Z_02	X_ZP_02	X_ZPS_02
$W_{10}^{d}$—energy absorption for 10% strain, (kJ/m^3^)	55.88 (1.00)	154.68 (2.77)	72.63 (1.30)	95.61 (1.71)
$W_{\mathrm{eff}.10}^{d}$—energy absorption efficiency for 10% strain, (%)	53.92 (1.00)	88.99 (1.65)	51.61 (0.96)	77.77 (1.44)
$W_{20}^{d}$—energy absorption for 20% strain, (kJ/m^3^)	199.39 (1.00)	344.98 (1.73)	256.28 (1.29)	233.56 (1.17)
$W_{\mathrm{eff}.20}^{d}$—energy absorption efficiency for 20% strain, (%)	54.74 (1.00)	84.60 (1.55)	59.10 (1.08)	74.94 (1.37)
$W_{30}^{d}$—energy absorption for 30% strain, (kJ/m^3^)	420.02 (1.00)	558.25 (1.33)	491.86 (1.17)	414.63 (0.99)
$W_{\mathrm{eff}.30}^{d}$—energy absorption efficiency for 30% strain, (%)	53.80 (1.00)	82.79 (1.54)	63.68 (1.18)	64.88 (1.21)
$W_{40}^{d}$—energy absorption for 40% strain, (kJ/m^3^)	726.63 (1.00)	793.89 (1.09)	782.13 (1.08)	668.77 (0.92)
$W_{\mathrm{eff}.40}^{d}$—energy absorption efficiency for 40% strain, (%)	50.48 (1.00)	81.06 (1.61)	58.25 (1.15)	55.51 (1.10)
$W_{\mathrm{eff}}^{d.\max}$—maximum energy absorption efficiency, (%)	54.77 (1.00)	90.10 (1.65)	63.72 (1.16)	81.55 * (1.49) 75.98 (1.39)
Strain for the maximum energy absorption efficiency, (%)	17.95 (1.00)	8.30 (0.46)	31.29 (1.74)	11.63 * (0.65) 20.41 (1.14)

* Becasue two distinct local maxima of energy absorption efficiency were visible in the energy absorption efficiency for specimen X_ZPS_02, they are both given in the table, along with the respective strain values for which each of them occurred.

**Table 6:** Characteristic measures determined in the cyclic dynamic tests.

Measure	X_P_03	X_Z_03	X_ZP_03	X_ZPS_03	X_ZPS_05_V
$\sigma_{\max}—$maximum stress after which the initial unloading started, (MPa)	0.91	2.21	1.26	1.49	1.50
$q_{W.\mathrm{el}}^{\mathrm{tot}}$—potential elastic strain energy share quotient after 10 loops, (%)	75.1	27.2	64.4	64.0	56.1
$q_{W.\mathrm{diss}}^{\mathrm{tot}}$—dissipated energy share quotient after 10 loops, (%)	24.8	72.7	35.5	35.9	43.8
$q_{W.\mathrm{diss}}^{\;\mathrm{norm}.\mathrm{tot}}$—quotient of accumulated normalized dissipated energy after 10 loops, (%)	584.4	101.6	517.2	387.0	212.4

## Data Availability

Not applicable.
